# Supplementary material for: Distribution, ecological risk assessment and source identification of pollutants in soils of different land-use types in degraded wetlands
Source: PeerJ. 2022 Feb 22;10:e12885. doi: 10.7717/peerj.12885 (PMC8877397; doi:10.7717/peerj.12885)
Supplement: Supplemental Information 2 [file peerj-10-12885-s002.zip › PCA Script.docx]

library(readxl)

library(factoextra)

library(FactoMineR)

df.pca<-read_xls("/Users/1107/Data.xls")

df.pca<-as.data.frame(df.pca)

rownames(df.pca)<-df.pca$Site

df.pca$Site <-NULL

res.pca <- PCA(df.pca, graph = FALSE)

eig.val <- get_eigenvalue(res.pca)

fviz_eig(res.pca, addlabels = TRUE, ylim = c(0, 50))

site<-c(rep("N",10),rep("H",6),rep("D",9))

fviz_pca_biplot(res.pca,

col.ind = site, palette = "jco",

addEllipses = TRUE, label = "var",

col.var = "black", repel = TRUE,

legend.title = "Site")
