# Supplementary material for: Distribution, ecological risk assessment and source identification of pollutants in soils of different land-use types in degraded wetlands
Source: PeerJ. 2022 Feb 22;10:e12885. doi: 10.7717/peerj.12885 (PMC8877397; doi:10.7717/peerj.12885)
Supplement: Supplemental Information 3 [file peerj-10-12885-s003.docx]

**Table S1** Classification of Nemerow pollution index

| **Degree** | ***P*_TN_** | ***P*_TP_** | ***P_n_*** | **Level** |
| --- | --- | --- | --- | --- |
| 1 | *P*_TN_≤1.0 | *P*_TP_≤0.70 | *P_n_*≤0.70 | Clean |
| 2 | 1.0＜*P*_TN_≤2.0 | 0.7＜*P*_TP_≤1.0 | 0.7＜*P_n_*≤1.0 | General clean |
| 3 | 2.0＜*P*_TN_≤3.0 | 1.0＜*P*_TP_≤2.0 | 1.0＜*P_n_*≤2.0 | Light pollution |
| 4 | 3.0＜*P*_TN_≤5.0 | 2.0＜*P*_TP_≤3.0 | 2.0＜*P_n_*≤3.0 | Moderate pollution |
| 5 | *P*_TN_＞5.0 | *P*_TP_＞3.0 | *P_n_*＞3.0 | Heavy pollution |
