# Supplementary material for: Distribution, ecological risk assessment and source identification of pollutants in soils of different land-use types in degraded wetlands
Source: PeerJ. 2022 Feb 22;10:e12885. doi: 10.7717/peerj.12885 (PMC8877397; doi:10.7717/peerj.12885)
Supplement: Supplemental Information 4 [file peerj-10-12885-s004.docx]

**Table S2** Classification of sediment *I_geo_* pollution degree

| Range | *I_geo_*≤0 | 0＜*I_geo_*≤1 | 1＜*I_geo_*≤2 | 2＜*I_geo_*≤3 | 3＜*I_geo_*≤4 | 4＜*I_geo_*≤5 | 5＜*I_geo_*≤6 |
| --- | --- | --- | --- | --- | --- | --- | --- |
| Level | Unpolluted | Low polluted | Moderately polluted | Moderately to strongly polluted | Strongly polluted | Strongly to very strongly polluted | Very strongly polluted |
