# Supplementary material for: Distribution, ecological risk assessment and source identification of pollutants in soils of different land-use types in degraded wetlands
Source: PeerJ. 2022 Feb 22;10:e12885. doi: 10.7717/peerj.12885 (PMC8877397; doi:10.7717/peerj.12885)
Supplement: Supplemental Information 5 [file peerj-10-12885-s005.docx]

**Table S3** Classification of pollution degree of the potential ecological risk index of sediment

| $E_{r}^{i}$ | Risk level | *RI* | Risk level |
| --- | --- | --- | --- |
| $E_{r}^{i}$＜40 | Low | *RI*＜150 | Low |
| 40≤$E_{r}^{i}$＜80 | Medium | 150≤*RI*＜300 | Medium |
| 80≤$E_{r}^{i}$＜160 | Higher than medium | 300≤*RI*＜600 | Higher than medium |
| 160≤$E_{r}^{i}$＜320 | High | *RI*≥600 | High |
| $E_{r}^{i}$≥320 | Very high | — | — |
