# Supplementary material for: Distribution, ecological risk assessment and source identification of pollutants in soils of different land-use types in degraded wetlands
Source: PeerJ. 2022 Feb 22;10:e12885. doi: 10.7717/peerj.12885 (PMC8877397; doi:10.7717/peerj.12885)
Supplement: Supplemental Information 6 [file peerj-10-12885-s006.docx]

**Table S4** Multivariate test of multivariate analysis of variance

|  | | Value | F | Assumed degrees of freedom | Error degrees of freedom | *p* |
| --- | --- | --- | --- | --- | --- | --- |
| Intercept | Pillai's trace | 0.996 | 285.250 | 11.000 | 12.000 | ＜0.001 |
|  | Wilks'Lanbda | 0.004 | 285.250 | 11.000 | 12.000 | ＜0.001 |
|  | Hotelling's trace | 261.480 | 285.250 | 11.000 | 12.000 | ＜0.001 |
|  | Roy's largest root | 261.480 | 285.250 | 11.000 | 12.000 | ＜0.001 |
| Land-use type | Pillai's trace | 1.504 | 3.582 | 22.000 | 26.000 | 0.001 |
|  | Wilks'Lanbda | 0.046 | 4.001 | 22.000 | 24.000 | 0.001 |
|  | Hotelling's trace | 8.807 | 4.403 | 22.000 | 22.000 | ＜0.001 |
|  | Roy's largest root | 7.127 | 8.422 | 11.000 | 13.000 | ＜0.001 |
