# Supplementary material for: Distribution, ecological risk assessment and source identification of pollutants in soils of different land-use types in degraded wetlands
Source: PeerJ. 2022 Feb 22;10:e12885. doi: 10.7717/peerj.12885 (PMC8877397; doi:10.7717/peerj.12885)
Supplement: Supplemental Information 7 [file peerj-10-12885-s007.docx]

**Table S5** Comprehensive pollution index and classification of nitrogen and phosphorus in surface sediments

| Site | H1 | H2 | H3 | H4 | H5 | H6 | D1 | D2 | D3 | D4 | D5 | D6 | D7 | D8 | D9 |
| --- | --- | --- | --- | --- | --- | --- | --- | --- | --- | --- | --- | --- | --- | --- | --- |
| *P_TN_* | 5.56 | 1.32 | 2.22 | 1.36 | 2.96 | 4.02 | 5.22 | 8.69 | 5.42 | 8.60 | 7.65 | 7.07 | 7.44 | 7.67 | 2.91 |
| Degree | 5 | 2 | 3 | 2 | 3 | 4 | 5 | 5 | 5 | 5 | 5 | 5 | 5 | 5 | 3 |
| *P_TP_* | 1.35 | 1.29 | 1.55 | 1.88 | 1.23 | 1.83 | 1.65 | 1.7 | 1.47 | 1.67 | 1.57 | 1.42 | 1.65 | 2.35 | 1.18 |
| Degree | 3 | 3 | 3 | 3 | 3 | 3 | 3 | 3 | 3 | 3 | 3 | 3 | 3 | 4 | 3 |
| *P_n_* | 4.63 | 1.31 | 2.06 | 1.76 | 2.57 | 3.51 | 4.42 | 7.16 | 4.54 | 7.08 | 6.32 | 5.83 | 6.16 | 6.48 | 2.51 |
| Degree | 5 | 3 | 4 | 3 | 4 | 5 | 5 | 5 | 5 | 5 | 5 | 5 | 5 | 5 | 4 |

Note: Grades 1 to 5 are clean, general clean, light pollution, moderate pollution, and heavy pollution.
