# Supplementary material for: Distribution, ecological risk assessment and source identification of pollutants in soils of different land-use types in degraded wetlands
Source: PeerJ. 2022 Feb 22;10:e12885. doi: 10.7717/peerj.12885 (PMC8877397; doi:10.7717/peerj.12885)
Supplement: Supplemental Information 8 [file peerj-10-12885-s008.docx]

**Table S6** Varimax rotated component matrix (the KMO= 0619, and the significance of Bartlett’s sphericity test is <0.001).

| Eigenvalues | 4.38 | 2.41 | 1.33 | 1.13 |
| --- | --- | --- | --- | --- |
| Variance (%) | 39.8 | 22.1 | 12.1 | 10.2 |
| Cummulative (%) | 39.8 | 61.9 | 74.0 | 84.2 |
| **Variable** | **PC1** | **PC2** | **PC3** | **PC4** |
| Pb | 0.83 | -0.01 | -0.11 | 0.38 |
| Cd | 0.09 | 0.05 | 0.04 | 0.96 |
| Zn | 0.74 | 0.29 | 0.50 | 0.24 |
| Cu | 0.92 | 0.10 | 0.26 | -0.08 |
| Cr | 0.07 | -0.21 | 0.89 | -0.11 |
| Ni | 0.80 | -0.10 | 0.32 | -0.08 |
| As | 0.88 | -0.16 | 0.06 | 0.09 |
| Hg | 0.32 | 0.26 | 0.67 | 0.21 |
| TN | -0.08 | 0.97 | 0.03 | 0.05 |
| TP | 0.49 | 0.55 | -0.22 | -0.18 |
| OM | -0.08 | 0.97 | 0.04 | 0.09 |
