# Supplementary figures and images for: Distribution, ecological risk assessment and source identification of pollutants in soils of different land-use types in degraded wetlands
Source: PeerJ. 2022 Feb 22;10:e12885. doi: 10.7717/peerj.12885 (PMC8877397; doi:10.7717/peerj.12885)

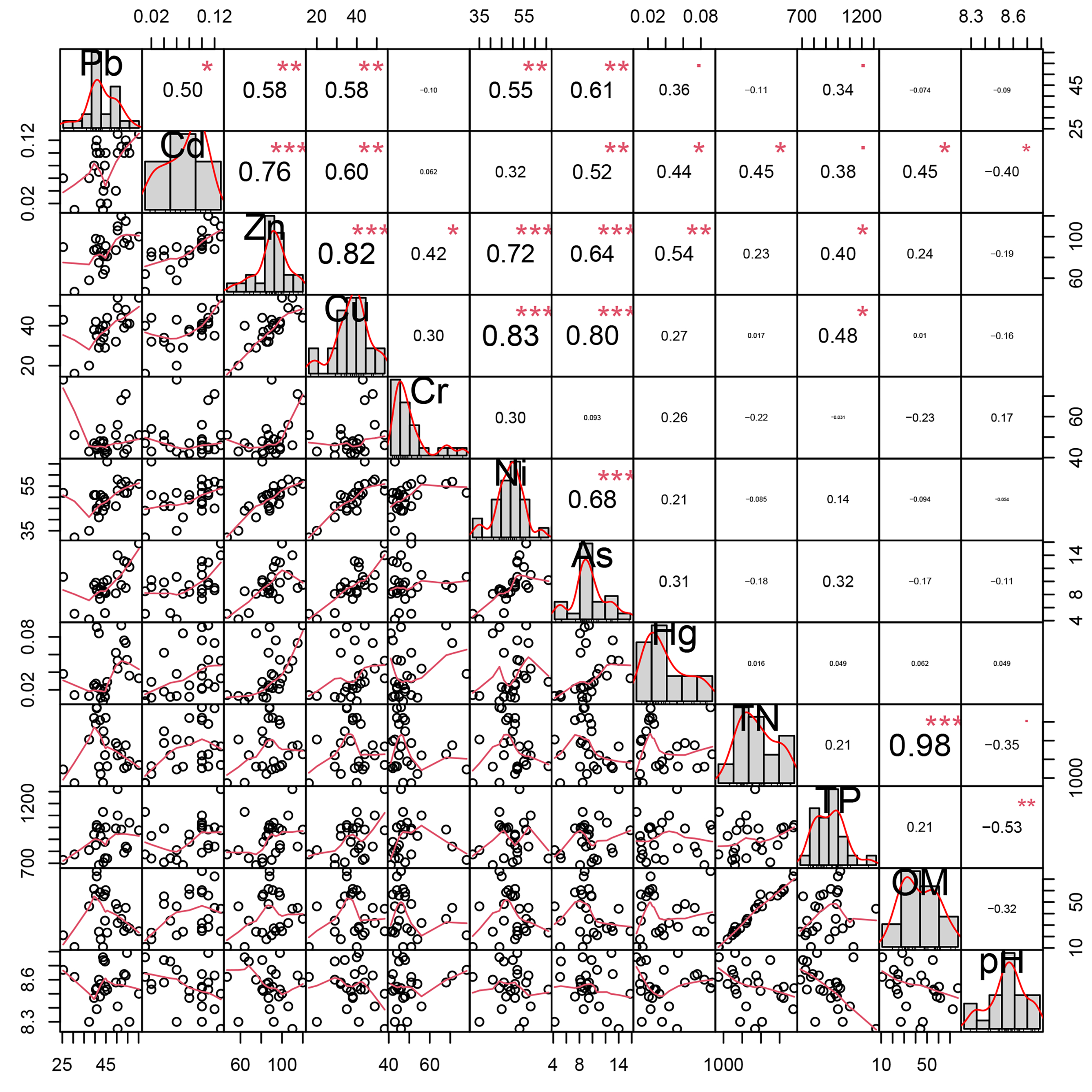

Supplement: Supplemental Information 9 [file peerj-10-12885-s009.png]
